# The relationship between career adaptability, job search self-efficacy, and EFL college students’ employment anxiety: the perspective of proactive personality

**DOI:** 10.3389/fpsyg.2026.1842585

**Published:** 2026-07-22

**Authors:** Su Zou, Lisheng Liu

**Affiliations:** School of Foreign Languages, Xuchang University, Xuchang, Henan, China

**Keywords:** career adaptability, EFL college students, employment anxiety, job search self-efficacy, proactive personality

## Abstract

**Background:**

In the current era where career prospects are highly uncertain, EFL college students generally encounter the real-life predicament of employment anxiety. Although existing research has devoted some attention to the issue of employment anxiety, our understanding of the underlying psychological mechanisms involved in alleviating this anxiety remains limited. To address this, this study drew on Social Cognitive Career Theory to look at how career adaptability relates to employment anxiety in EFL college students. It also explored the mediating role of job search self-efficacy and the moderating role of proactive personality.

**Method:**

This study collected data from 916 EFL college students using stratified convenient sampling. Participants completed the survey through an online platform. Path analysis was conducted using the PROCESS macro in SPSS.

**Results:**

The results show that career adaptability is significantly negatively correlated with employment anxiety. Job search self-efficacy partially mediates this relationship, accounting for 33.78% of the total effect. Students with higher proactive personality scores are better able to use career adaptability and job search self-efficacy to cope with employment anxiety. Inter-group difference analysis indicates that EFL college students of different genders and grades have significant differences in employment anxiety.

**Conclusion:**

This study offers the deeper understanding of how internal resources and positive personality traits interact to help people cope with employment anxiety. These findings offer the empirical basis for the optimization of mental health education and employment guidance services in colleges and universities.

## 1 Introduction

Employment anxiety is the tension, worry, or even fear people feel when they are unsure whether their skills match the demands of the job market, when their career path seems uncertain, or when the pressure of competition begins to weigh on them (Wang and Zhang, 2025). As the job hunting for college graduates becomes increasingly difficult, this kind of anxiety will only become more and more widespread. One survey of 719 Chinese college students found that employment anxiety is now a widespread issue among students. It closely tied to their mental health and how well they can adapt to their careers (Wang G. J. et al., 2025). Previous studies have linked employment anxiety to a range of mental health issues. Students experiencing such anxiety often report physical symptoms such as poor sleep or chest tightness. Beyond these health concerns, research also suggests that employment anxiety can interfere with career planning, which may affect students’ long-term professional development (Wu et al., 2024; Yang et al., 2025). Within this context, EFL college students face a more complex employment dilemma. Unlike many applied or technically-oriented majors, the training model for foreign language majors has long centered on linguistic skills and humanistic literacy (Harbuza et al., 2025). However, the current job market increasingly demands talent that combines foreign language proficiency with other professional skills. This structural mismatch makes EFL college students more likely to perceive a gap between their capabilities and job requirements during their job search, thereby exacerbating their employment anxiety. Career adaptability has gained more attention in the literature recently. Scholars tend to see it as a kind of psychological resource, one that helps people navigate career-related challenges and cope with uncertainty. For university students, this resource may offer a greater sense of control when they are looking for a job, which in turn could help reduce employment anxiety (Fang and Xu, 2025). Yet adaptability resources alone may not tell the full story. There are likely other mechanisms at play in how employment anxiety develops, and how it can be eased. Job search self-efficacy (JSSE), that is, their subjective assessment of their ability to successfully accomplish this task. It helps them respond to emerging difficulties with greater calm and composure, which significantly reduces the level of anxiety associated with job hunting (Carkit, 2025). In addition, an individual’s behavioral tendencies also play a significant role in this. Proactive personality, a relatively stable trait, may also matter. People who exhibit this trait to a significant degree typically seek out information, ask for support, and adjust their strategies as needed. This further enhances its adaptability in stressful situations, becoming an important protective factor in resisting employment anxiety (Wan et al., 2024). Therefore, a thorough examination of coping mechanisms related to employment anxiety is of great theoretical and practical importance for the development of effective psychological interventions and for facilitating a smooth transition for students from educational institutions to the workforce.

Existing studies have shown that career adaptability, JSSE, and proactive personality can each play positive roles in career development. Most studies tend to test the independent contributions of the above variables and rarely incorporate them into a unified framework to systematically examine their synergistic mitigation mechanism on college students’ employment anxiety. In particular, there remains a lack of sufficient empirical testing regarding how career adaptability relates to employment anxiety through psychological beliefs in the job-seeking context, as well as the role that individual proactive traits play within this relational network. To address these questions, this study is based on Social Cognitive Career Theory (SCCT) (Lent et al., 1994). From an integrated perspective, it looks at how career adaptability, JSSE and proactive personality come together in the employment anxiety situation of college students. Given the particular challenges that EFL students face, both in their training and in the job market’s expectations, this study focuses on their experience. The goal is to offer both theoretical insights and practical suggestions for reducing employment anxiety in this group, and for rethinking how academic programs prepare students for careers.

## 2 Literature review and hypotheses

### 2.1 Theoretical basis

SCCT emphasizes that people’s career development arises from the interaction among personal background, self-efficacy, and environmental factors (Lent et al., 1994). For EFL college students, the uncertainty of the macro-level employment situation and intensified competition for positions matching their majors often expose them to varying degrees of employment anxiety. For students facing career challenges, career adaptability can help them maintain psychological stability and proactively adjust their behavior during career transitions and uncertain environments, thereby alleviating to a certain extent the anxiety that may arise in the context of employment pressure (Boo et al., 2021). Whether this protective effect actually works may depend on how much confidence people have in their own ability to find a job. Individuals with higher career adaptability are more capable of flexibly responding to changes in the occupational environment and maintaining confidence in their own abilities when encountering setbacks in job hunting, that is, they have higher JSSE (Matijas and Sersic, 2021). And a strong sense of self-efficacy serves as an important psychological foundation for overcoming anxiety. This helps them develop more confidence in shaping their careers, so that pressure becomes an obstacle they can overcome rather than something that overwhelms them (Li R. H. et al., 2025). In addition, college students exhibiting high proactivity are better at transforming vocational adaptability into positive efficacy cognition (Meng et al., 2025). This will further alleviate employment anxiety more effectively. Based on the theoretical principles outlined above, the main reason for focusing on the group of EFL college students is that this group finds itself in a situation where macro-level pressures from the labor market intersect with micro-level individual psychological resources. This investigation can provide empirical evidence for optimizing the psychological support system within foreign language talent cultivation.

### 2.2 Career adaptability and employment anxiety

Career adaptability, in simple terms, refers to people’s ability to actively adjust their knowledge, skills, attitudes, and working methods during the process of career development. They can flexibly respond to changes in the workplace and meet the requirements of the position, thereby promoting the continuous advancement of their career (Savickas, 1997). From the perspective of SCCT (Lent et al., 1994), career adaptability is a key psychological resource for college students when facing employment pressure. It not only helps college students establish a sense of career identity from multiple aspects, but also enables people to better control environmental changes and have more positive expectations for career development. As a result, it can effectively alleviate the psychological pressure caused by the contradictions in the employment market structure. Research has indicated that career adaptability and employment anxiety are closely linked, specifically tending to be negatively correlated (Fang and Xu, 2025). In other words, when a person has strong career adaptability, they tend to generate more stable and lasting career development motivation (Lin et al., 2024), and at the same time, they will perform better in terms of the clarity of career direction and confidence in dealing with problems. All these help reduce the anxiety caused by uncertainty during career development (Alexander et al., 2025). For instance, a study by Dong et al. (2023) indicated that career adaptability can buffer the impact of career pressure, thereby reducing an individual’s risk of experiencing anxiety. Based on this, the present study focuses on career adaptability and employment anxiety in EFL college students. It aims to explore the unique employment challenges faced by this group in the context of language majors and their psychological coping mechanisms. Mapping out the interaction pathway between the two supplies a theoretical base and practical methods for designing professional support and counseling services that fit this group’s particular requirements.

### 2.3 The mediating role of job search self-efficacy

JSSE refers to the degree of subjective certainty and confidence that an individual holds regarding their ability to effectively complete necessary actions and achieve employment goals when dealing with job-related tasks (Saks, 2006). SCCT (Lent et al., 1994) considers this to be an important cognitive factor that drive individual career behavior. When individuals demonstrate high career adaptability, their internal belief system often plays a more positive role in alleviating employment anxiety and enhancing psychological resilience in uncertain situations. A recent meta-analysis on JSSE, through systematic examination of prior literature, reports a positive association between career adaptability and this construct. This finding shows high consistency in multiple empirical studies (Zheng et al., 2025). More precisely, higher career adaptability among college students tends to co-occur with stronger JSSE (Gerçek, 2024). This suggests that college students with greater career adaptability are more capable of actively mobilizing environmental resources and their own strengths, thereby maintaining higher JSSE and holding more positive expectations for their career development (Al-Jubari et al., 2021). Research by Morici et al. (2022) also shows that cultivating career adaptability represents an effective approach to strengthening individuals’ confidence and enhancing their JSSE. Therefore, systematically improving the career adaptability can help students build a more stable and resilient foundation of confidence in their job searches.

There’s a fair amount of evidence linking self-efficacy to employment anxiety. Liu and Zhang (2025), for instance, showed that college students with stronger self-efficacy tended to experience lower employment anxiety. One explanation comes from Li R. H. et al. (2025), who suggest that self-efficacy gives people the feeling of control over the job search process, and that sense of control can help offset the anxiety that uncertainty brings. Gerçek and Özveren (2025) reached a similar conclusion in their work on college students and career anxiety. There’s also some experimental evidence. Escher et al. (2025) showed that targeted training programs can boost JSSE, and in doing so, help lower anxiety. Additionally, some research suggests that JSSE serves as an important mediator between job search support and employment anxiety (Carkit, 2025). But when it comes to the relationship between career adaptability and employment anxiety, especially among EFL students. We still do not know much about how JSSE comes into play. Empirical work in this area is sparse. Based on this, this study focuses on EFL college students, aiming to investigate and verify the mediating mechanism of JSSE between career adaptability and employment anxiety, with the expectation of filling the research gap in this field and providing support for related psychological intervention.

### 2.4 The moderating role of proactive personality

Proactive personality is a term Bateman and Crant (1993) introduced to describe people who tend to take initiative. They take the initiative to act to intervene in or transform the environment instead of passively adapting to it. SCCT (Lent et al., 1994) emphasizes that individuals do not passively accept the influence of the environment, but can actively regulate their cognitive processes and behavioral outcomes through their own initiative. From this theoretical perspective, the proactive personality holds significant driving significance in the individual’s career development process. This element encompasses cognitive interpretations of the career environment, self-efficacy perceptions, and the ways adverse conditions relate to employment psychology within this population. Prior research notes a close link between proactive personality and college students’ job-preparation activities, with this trait also appearing alongside career exploration and employment adjustment efforts (Ruan et al., 2025). For instance, the proactive personality has a promoting role on college students’ career adaptability in China (Ling et al., 2022), and it is also positively correlated with their self-efficacy demonstrated in the career development (Zhang et al., 2024). Moreover, when facing employment pressure, individuals with a higher proactivity experience relatively lower psychological impacts (Tu and Liu, 2025). The proactive personality can also reduce the anxiety experienced by college students in the employment process by enhancing their self-efficacy (Feng and Zhang, 2025). At the same time, Wang J. J. et al. (2025) identified proactive personality as a factor that relates to the connection between career adaptability and career outcome expectations. In summary, the proactive personality demonstrates prominent protective functions in enhancing career adaptability, maintaining JSSE, and alleviating employment anxiety. However, existing studies largely treat it as a direct antecedent variable, whereas its function as a moderating factor and the circumstances under which it operates have received comparatively less focus. Accordingly, this study positions proactive personality as the focal trait for examining its moderating role in relation to employment anxiety and the associated boundary conditions.

### 2.5 This study

**Figure 1:**
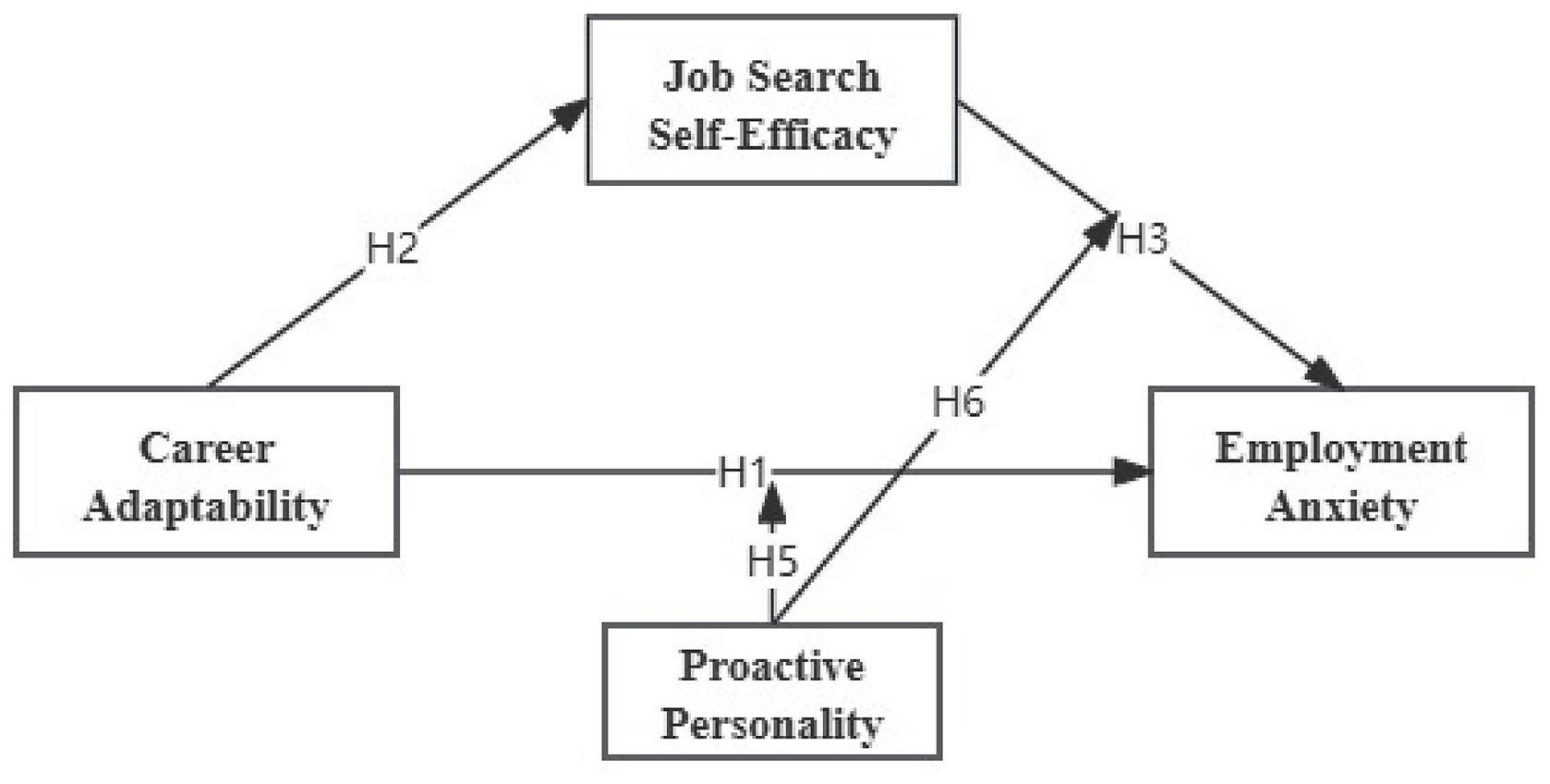
Hypothesis model.

This study adopts SCCT as its theoretical foundation for describing the associations among career adaptability, JSSE, proactive personality, and employment anxiety in EFL college students. Our investigation focused on two principal aspects: the mediating function of JSSE and the moderating role of proactive personality. Based on the interactive mechanisms of individual cognition, behavior, and environmental factors emphasized by SCCT, this study posits that career adaptation, as a key resource for individuals coping with career transitions, may be positively associated with JSSE, and together they may be linked to lower employment anxiety; meanwhile, proactive personality may moderate the relationships of career adaptation and JSSE with employment anxiety. Based on this, this study constructs a moderated mediation model (as shown in Figure 1) and proposes the following hypotheses:

*H1*: Career adaptability is significantly negatively correlated with employment anxiety among EFL college students.

*H2*: Career adaptability is significantly positively correlated with JSSE among EFL college students.

*H3*: JSSE is significantly negatively correlated with employment anxiety among EFL college students.

*H4*: JSSE mediates the relationship between career adaptability and employment anxiety.

*H5*: Proactive personality moderates the relationship between career adaptability and employment anxiety.

*H6*: Proactive personality moderates the relationship between JSSE and employment anxiety.

## 3 Methods

### 3.1 Participants and data collection

The data collection for this study was conducted from February to March in 2026 using an online questionnaire platform^1^. Participants were recruited through convenience sampling from multiple universities in Henan province. The questionnaire link was distributed via university cooperation networks, class groups, and social media platforms. To ensure adequate demographic heterogeneity, recruitment intentionally targeted diverse grade levels and age groups. Regarding gender balance, we implemented systematic efforts during recruitment to avoid disproportionate representation. Specifically: (a) the questionnaire link was distributed to classes across multiple disciplines (e.g., humanities, sciences, and engineering) rather than a single major; (b) we asked course instructors and class monitors to forward the link to both male and female students equally; and (c) we monitored the gender ratio of incoming responses on a daily basis and made supplementary distributions to male-dominated classes when female responses were overrepresented. As a result, the final sample achieved a reasonably balanced gender composition (439 male, 477 female). Following established practices in theory-testing research (Highhouse and Gillespie, 2010), we adopted convenience sampling given that the primary aim of this study is to examine hypothesized relationships among constructs rather than to estimate population parameters. We incorporated gender, age, and grade as covariates across all regression models to address possible selection-related distortions (Becker et al., 2016). Before participation, each respondent received a full description of the study’s aims and steps, after which written consent was secured. Taking part was completely optional, and participants were told they could stop whenever they wished. The questionnaire was completed without identifying information, and all responses were kept in a protected database. The research plan was approved by the University Ethics Review Committee.

The sample size was estimated based on the method proposed by Kline (2018). It stipulates that each question should have at least 10 respondents. The total number of questions in this survey was 75. Considering that there might be 20% of invalid questionnaires or dropouts, the minimum number of samples required was calculated to be 900. In fact, 945 questionnaires were sent out, and the response rate was 100%. After the questionnaires were collected, we conducted a strict screening process, and the criteria for data cleaning included: (1) Valid responses required completion of at least 80% of the total items. Those with over 20% missing values were excluded from analysis. For cases containing partial missing data below the 20% threshold, multiple imputation was applied to address the missing entries; (2) If the answers showed obvious extreme reaction tendencies, such as all selecting “strongly disagree” or “strongly agree,” such questionnaires would be excluded; (3) Referring to the work of Meade and Craig (2012), the lower limit for the 3rd percentile of the response time for all participants (70 s) was used as the benchmark; those with a response time lower than this would be directly excluded. The final dataset comprised 916 valid questionnaires, representing a valid return rate of 96.93%. The distribution of the basic demographic characteristics of the participants is shown in Table 1. The sample was distributed reasonably on key variables and could meet the requirements for subsequent analysis.

**Table 1:** Distribution of demographic characteristics.

Demographic characteristic variables	Category	Amount	Percentage (%)
Gender	Male	439	47.93
	Female	477	52.07
Age	18-19 years old	341	37.23
	20-21 years old	332	36.24
	22 years old and above	243	26.53
Grade	Freshman	233	25.44
	Sophomore	266	29.04
	Junior	228	24.89
	Senior	189	20.63

### 3.2 Measurement instruments

#### 3.2.1 Career adaptability

This study assessed career adaptability using the Career Adapt-Abilities Scale developed by Savickas and Porfeli (2012) and revised by Hou et al. (2012). This scale comprises 24 items (e.g., “I look for opportunities to grow and develop”), encompassing four dimensions: concern, control, curiosity, and confidence. This instrument uses a 5-point Likert scale. Higher scores indicate higher level of career adaptability. This scale demonstrates good validity among Chinese college students (Ouyang et al., 2025). The standardized factor loadings for the 24 items ranged from 0.53 to 0.85.

#### 3.2.2 Job search self-efficacy

This study assessed JSSE using the scale developed by Saks et al. (2015) with 20 items. A sample question is: “I am confident I can find a job soon.” It encompasses two dimensions: job search behavior self-efficacy and job search outcome self-efficacy. This instrument uses a 5-point Likert scale. Higher score means students have greater JSSE. This scale demonstrates good validity among Chinese college students (Guan et al., 2022). The standardized factor loadings for the 20 items ranged from 0.51 to 0.82.

#### 3.2.3 Proactive personality

This study measured proactive personality using the scale developed by Bateman and Crant (1993) and revised by Shang and Gan (2009) with 11 questions. This instrument uses a 7-point Likert scale. Example questions include, “I am constantly searching for new ways to improve my life.” Higher scores indicate a more pronounced proactive personality in college students. The scale demonstrates good reliability and validity among Chinese college students (Liu et al., 2025). The standardized factor loadings for the 11 items ranged from 0.57 to 0.87.

#### 3.2.4 Employment anxiety

This study assessed employment anxiety levels in college students using the scale developed and validated by Wang C. et al. (2025). Reliability and validity tests indicate this instrument is suitable for Chinese college student populations. The scale consists of 17 items. It covers four dimensions: personal ability, knowledge application, career substitutability, and social relationships. A sample question is: “I worry that my professional knowledge is not as solid as others.” Items are rated on a 5-point Likert scale. Higher scores representing higher levels of employment anxiety. The standardized factor loadings for the 17 items ranged from 0.52 to 0.84.

### 3.3 Statistical analysis

We used OLS-based path analysis with the PROCESS macro (Hayes, 2018) to test our moderated mediation model. Although full SEM with latent variables could account for measurement error, we selected PROCESS for three reasons: (1) it is specifically tailored for conditional process analysis and yields clear outputs for moderated mediation; (2) confirmatory factor analysis (CFA) results (see Section 4.2) demonstrated strong psychometric properties across all scales (CFI ≥ 0.967, factor loadings >0.50, Cronbach’s *α* > 0.93), supporting the use of composite scores; and (3) the regression-based approach enhances transparency and replicability in applied research.

The analysis proceeded as follows. First, we conducted descriptive statistics and assessed scale reliability and validity. Second, we performed CFA to evaluate measurement model fit and common method bias using AMOS. In the combined CFA model, all items for each construct were specified to load onto a single first-order factor to examine the global construct of each variable. This specification was supported by excellent model fit indices (see Section 4.2) and high internal consistency. Composite scores were computed as the arithmetic mean of all items per scale, following standard practice in career research (Hou et al., 2012). Third, we examined group differences in employment anxiety by gender (independent-samples *t*-test), age, and grade (one-way ANOVAs). Finally, we tested mediation (Model 4) and moderation (Model 15) using SPSS PROCESS 4.2, with bootstrap resampling (5,000 draws) to estimate 95% confidence intervals; intervals not containing zero indicated significant effects (Hayes and Rockwood, 2017).

## 4 Results

### 4.1 Descriptive statistics and correlation test

To ensure the data met the assumptions for multivariate analysis, this study verified data normality by examining skewness and kurtosis. We used means (*M*) and standard deviations (SD) for descriptive statistical analysis of variables that met the normality assumption. Following the criteria by Kim (2013), data were considered approximately normally distributed when the absolute skewness value was less than 2 and the absolute kurtosis value was less than 7. As shown in Table 2, all study variables satisfied these requirements for normal distribution.

**Table 2:** Correlation analysis and variable description.

Variables	*M* ± SD	Skewness	Kurtosis	Career adaptability	JSSE	Proactive personality	Employment anxiety
Career adaptability	3.568 ± 0.691	−0.605	−0.215	1			
JSSE	3.571 ± 0.762	−0.604	−0.343	0.589***	1		
Proactive personality	4.878 ± 1.204	−0.504	−0.381	0.550***	0.595***	1	
Employment anxiety	3.398 ± 0.714	−0.244	−0.201	−0.447***	−0.434***	−0.459***	1

*N* = 916, path significance: ***p < 0.001; JSSE, job search self-efficacy, the same below.

Table 2 shows that career adaptability is significantly related to other three variables in the expected directions: positively with JSSE and proactive personality, and negatively with employment anxiety. JSSE and proactive personality are strongly positive correlated, and JSSE is negatively associated with employment anxiety. Proactive personality and employment anxiety show a significant negative correlation. Taken together, these correlations were consistent with the theoretically predicted directions.

### 4.2 Measurement model and validity testing

Prior to examining the structural relationships among variables, we evaluated the overall measurement model through a combined CFA that included all four latent constructs (Career Adaptability, JSSE, Proactive Personality, and Employment Anxiety) simultaneously. The measurement model demonstrated excellent fit to the data: *χ*^2^/d*f* = 1.605, RMSEA = 0.026, SRMR = 0.032, GFI = 0.902, NFI = 0.917, CFI = 0.967, IFI = 0.967, TLI = 0.962, RFI = 0.906, all meeting or exceeding the recommended thresholds (Hair Jr et al., 2021; Hu and Bentler, 1998).

Both the Cronbach’s *α* coefficient and composite reliability (CR) values (see Table 3) were above the 0.7 benchmark (Hair Jr. et al., 2021). These findings indicate a high degree of internal consistency for the scales. All latent variables yielded average variance extracted (AVE) estimates exceeding the 0.5 criterion in the validity examination (Henseler et al., 2015), demonstrating good convergent validity. To assess discriminant validity, we applied the most stringent criteria. All heterotrait-monotrait (HTMT) ratios of correlations among the latent variables were below 0.85 (see Table 4). Table 5 further presents that the square roots of the AVE values surpassed the corresponding correlations with other variables for every construct. These two strict criteria mutually corroborated each other, collectively establishing the ideal discriminant validity of the scale (Hair Jr. et al., 2021).

**Table 3:** Reliability and validity.

Variables	Cronbach’s *α*	CR	AVE
Career adaptability	0.932	0.942	0.596
JSSE	0.943	0.949	0.522
Proactive personality	0.954	0.958	0.535
Employment anxiety	0.960	0.963	0.519

**Table 4:** The HTMT.

Variables	Career adaptability	JSSE	Proactive personality	Employment anxiety
Career adaptability				
JSSE	0.617			
Proactive personality	0.581	0.632		
Employment anxiety	0.470	0.457	0.488	

**Table 5:** The results of Fornell-Larcker criterion test.

Variables	Career adaptability	JSSE	Proactive personality	Employment anxiety
Career adaptability	**0.721**			
JSSE	0.594	**0.731**		
Proactive personality	0.551	0.598	**0.772**	
Employment anxiety	−0.451	−0.435	−0.461	**0.723**

Display the square root of the AVE values on the diagonal of the table in bold.

### 4.3 Common method bias (CMB)

All information for this study came from participant self-reports, which introduces the potential presence of CMB. For this purpose, we ran an initial check with Harman’s single-factor method. Exploratory factor analysis results showed six factors with eigenvalues above 1.0. The first factor explained 34.699 percent of the variance, under the 40 percent benchmark (Podsakoff et al., 2003), suggesting that common method bias did not pose a major issue here.

To enhance the robustness of this argument, we further employed the unmeasured latent method factor approach (Podsakoff et al., 2012). Following the establishment of the measurement model in Section 4.2, we used this model as the baseline model and constructed a control model by adding a common method factor, with the factor loadings from this common method factor to all items constrained to be equal. By comparing the fit between the two models, we examined the possible presence of common method bias. As shown in Table 6, both the baseline model and the control model exhibited satisfactory overall fit levels (Hu and Bentler, 1998). Evaluation standards for model comparisons were drawn from prior work on measurement invariance (Chen, 2007; Cheung and Rensvold, 2002), and all observed differences remained well below those recommended thresholds. These outcomes indicated that the addition of a common method factor did not produce a meaningful gain in model fit. Accordingly, CMB did not emerge as a substantial issue within the measurement model, which further aligned with the earlier observation from the Harman single-factor test.

**Table 6:** The comparison results between the baseline model and the control model.

Model	*χ*^2^/d*f*	RMSEA	SRMR	GFI	NFI	CFI	IFI	TLI	RFI
Reference value	<3	<0.06	<0.06	>0.9	>0.9	>0.9	>0.9	>0.9	>0.9
Baseline model	1.605	0.026	0.032	0.902	0.917	0.967	0.967	0.962	0.906
Control model	1.585	0.025	0.029	0.904	0.918	0.968	0.968	0.964	0.907
Difference	0.020	0.001	0.003	0.002	0.001	0.001	0.001	0.002	0.001
Judgment standard		Δ < 0.01	Δ < 0.025	Δ < 0.01	Δ < 0.01	Δ < 0.01	Δ < 0.01	Δ < 0.02	Δ < 0.01

### 4.4 Collinearity test

We examined potential multicollinearity using the variance inflation factor. All values in Table 7 were well below the threshold of 3 recommended by Hair Jr. et al. (2021), indicating that multicollinearity was not a concern in this study.

**Table 7:** The results of collinearity test.

Variables	Career adaptability	JSSE	Proactive personality	Employment anxiety
Career adaptability		1.000	1.727	
JSSE			1.703	
Proactive personality				
Employment anxiety			1.617	

### 4.5 Group differences

Independent samples t-tests were run to compare employment anxiety between male and female participants. One-way ANOVAs were used to examine variations across age groups and grade levels. As summarized in Table 8, significant differences were observed between the groups with respect to both gender and grade level. Female students had significantly higher employment anxiety than male students. Seniors also showed significantly greater anxiety than students in lower grades.

**Table 8:** Analysis result.

Outcome variable	Covariate		*M* ± SD	*T*/*F*	*p*
Employment anxiety	Gender	Male (*n* = 439)	3.129 ± 0.708	11.663	<0.001
		Female (*n* = 477)	3.646 ± 0.625		
	Age	18-19 years old (*n* = 341)	3.431 ± 0.704	0.716	0.489
		20-21 years old (*n* = 332)	3.365 ± 0.725		
		22 years old and above (*n* = 243)	3.398 ± 0.714		
	Grade	Freshman (*n* = 233)	3.342 ± 0.656	4.039	0.007
		Sophomore (*n* = 266)	3.327 ± 0.764		
		Junior (*n* = 228)	3.418 ± 0.641		
		Senior (*n* = 189)	3.543 ± 0.776		

### 4.6 Mediating effects analysis

Demographic variables were entered as control factors in the mediation model to account for possible confounding influences. Table 9 presents a significant negative correlation between career adaptability and employment anxiety, consistent with H1. Career adaptability was also positively associated with JSSE, while JSSE was negatively associated with employment anxiety. We examined the direct and total effects of career adaptability on employment anxiety, as well as the indirect effect through JSSE, with the full results presented in Table 10. The outcomes showed that JSSE served as a partial mediator in the career adaptability–employment anxiety relationship. These results are consistent with H2 through H4.

**Table 9:** Test of mediating role.

Outcome variable	Predictive variable	*β*	SE	*T*	95% CI		*R* ^2^	*F*
					LLCI	ULCI		
Employment anxiety	Career adaptability	−0.450	0.028	−16.309***	−0.504	−0.396	0.341	117.953
JSSE	Career adaptability	0.558	0.027	20.645***	0.505	0.611	0.367	132.096
Employment anxiety	Career adaptability	−0.298	0.032	−9.254***	−0.361	−0.235	0.623	115.330
	JSSE	−0.272	0.033	−8.331***	−0.336	−0.208		

**Table 10:** Path of mediating role.

Effect	Path	*β*	Percentage	95% CI		Intermediary type
				LLCI	ULCI	
Total effect	Career adaptability → employment anxiety	−0.450	100%	−0.504	−0.396	
Direct effect	Career adaptability → employment anxiety	−0.298	66.22%	−0.361	−0.235	
Indirect effect	Career adaptability → JSSE → employment anxiety	−0.152	33.78%	−0.194	−0.113	Partial mediation

### 4.7 Moderating effects analysis

**Figure 2:**
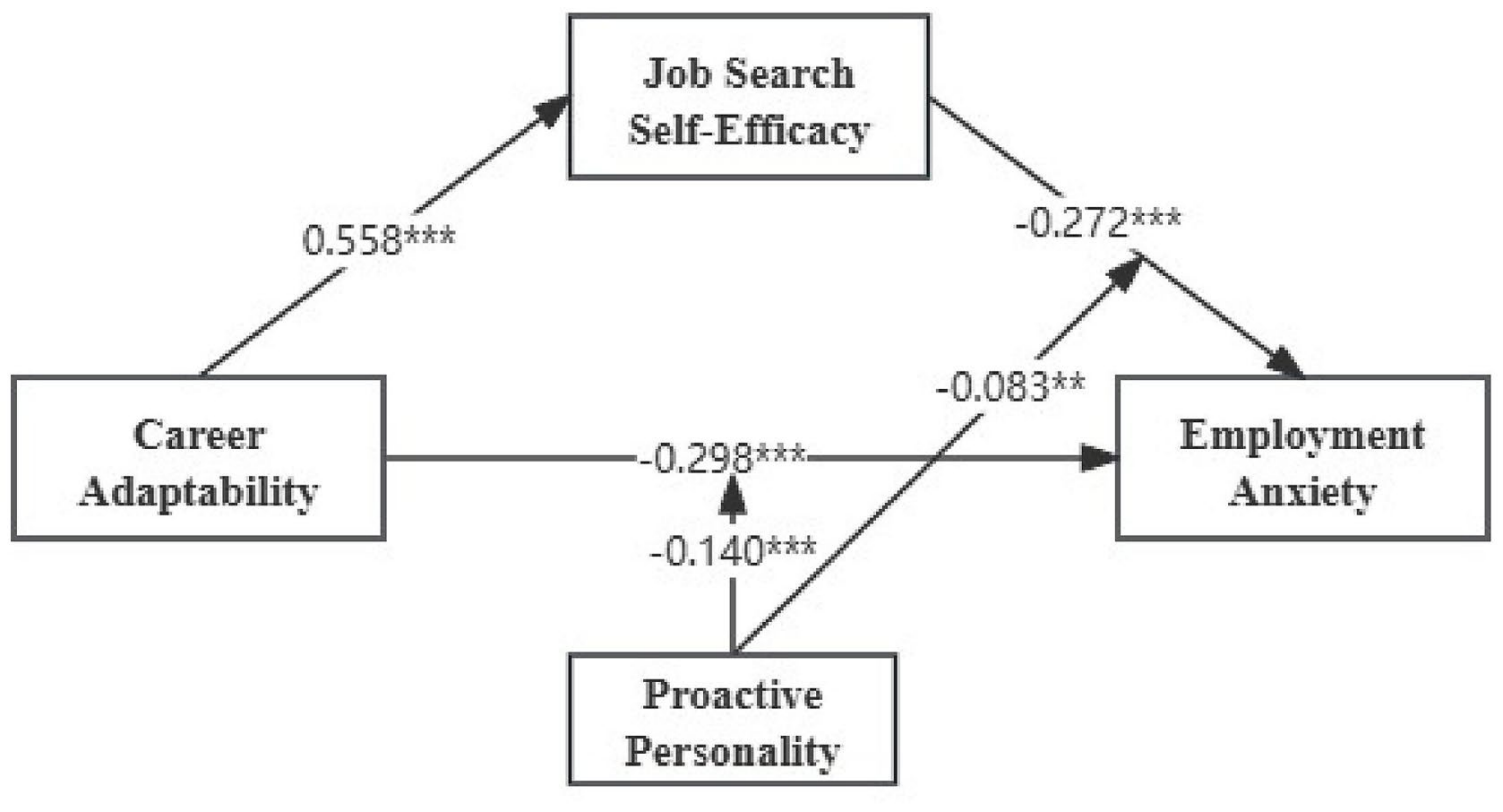
Path analysis diagram. Values on the paths represent standardized path coefficients. ***p < 0.001, **p < 0.01. Solid arrows indicate significant paths.

Table 11 presents the results of the moderation analysis. The interaction terms of proactive personality with career adaptability and with JSSE both showed significant negative associations with employment anxiety. These results indicate that proactive personality significantly moderates the relationships between career adaptability and employment anxiety, as well as between JSSE and employment anxiety. These findings support H5 and H6. Figure 2 illustrates the path analysis diagram.

**Table 11:** The moderating role of proactive personality.

Outcome variable	Predictive variable	*β*	*p*	SE	*T*	95% CI	
						LLCI	ULCI
Employment anxiety	Career adaptability × proactive personality	−0.140	<0.001	0.031	−4.519***	−0.201	−0.079
	JSSE × proactive personality	−0.083	0.009	0.032	−2.604**	−0.145	−0.020

Path significance: **p < 0.01.

### 4.8 Simple slope analyses

**Figure 3:**
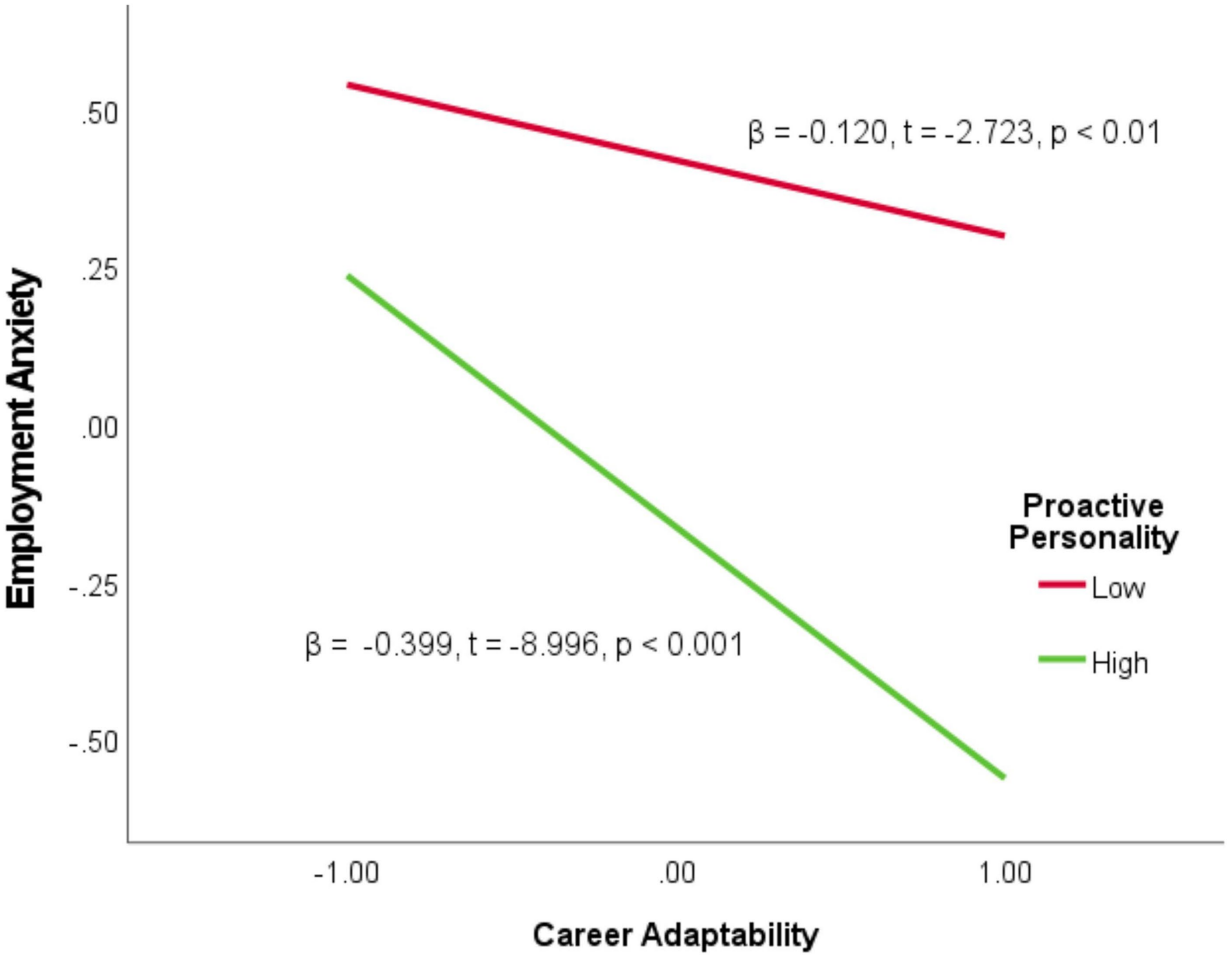
Simple slope analysis diagram of career adaptability and employment anxiety.

**Figure 4:**
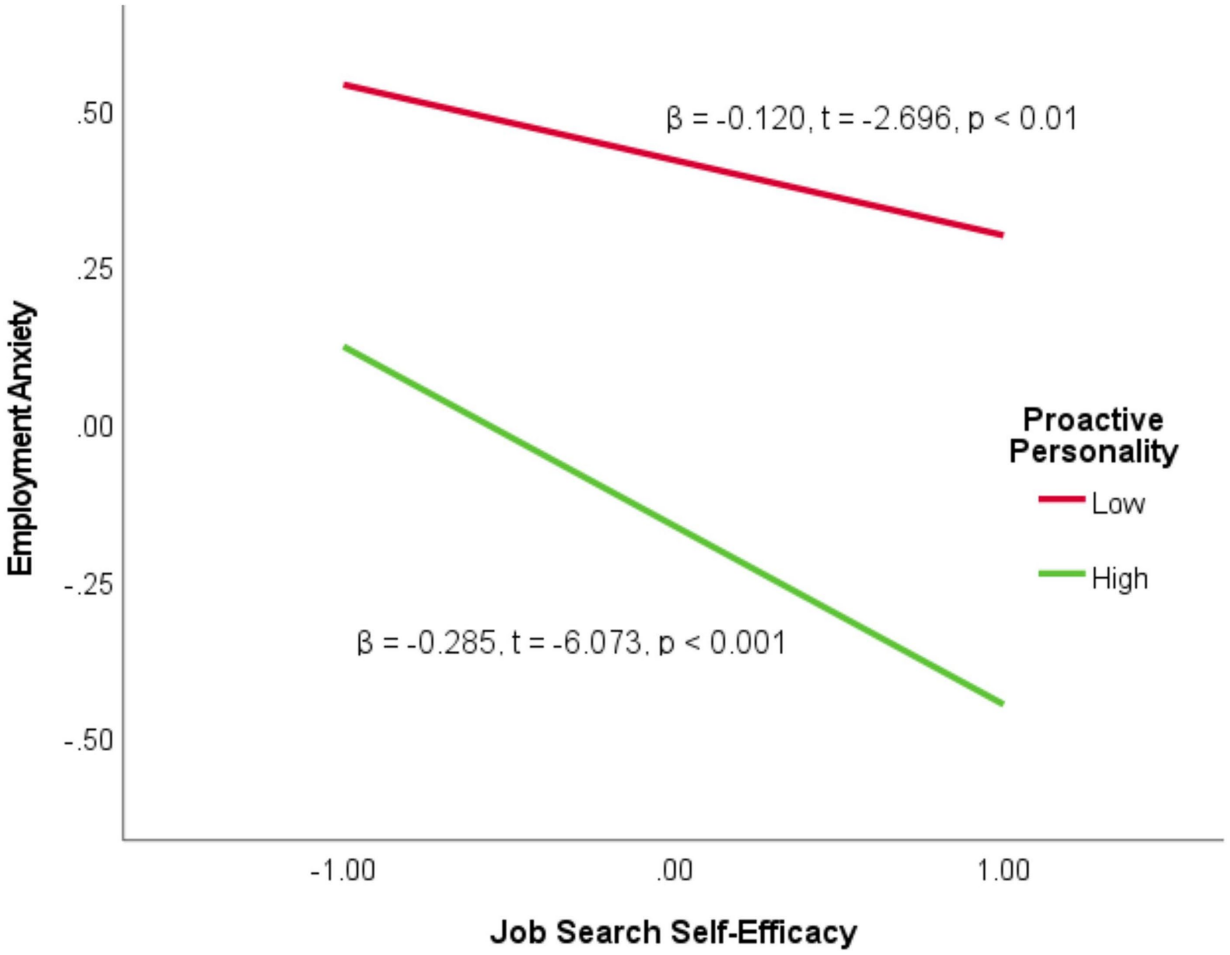
Simple slope analysis diagram of JSSE and employment anxiety.

To visualize the significant interactions and probe them further, we conducted simple slope analyses. For this purpose, participants were divided into low-score and high-score groups using one standard deviation below the mean (*M* − 1SD) and one standard deviation above the mean (*M* + 1SD) based on their proactive personality scores. As shown in Figures 3 and 4, for students with higher proactive personality, the negative association between career adaptability and employment anxiety was stronger. This leads to the conclusion that individuals with a highly proactive personality have a more prominent advantage effect in the process of career adaptation. A similar pattern emerged for JSSE. The negative relationship between JSSE and employment anxiety was more pronounced among students with high proactive personality. Individuals who score high on proactive personality tend to show greater engagement with their job-hunting self-efficacy in employment-related contexts. We applied the Johnson-Neyman method for additional examination of the moderating effects. Results indicated that when proactive personality scores exceeded a critical value (*Z* > −1.182), both career adaptability and JSSE significantly reduced employment anxiety. However, the moderating patterns differed across the two resources. As proactive personality decreased, the buffering effect of career adaptability became non-significant first, specifically within the range of −1.255 < *Z* < −1.182. When proactive personality fell below the lower threshold (*Z* < −1.255), neither resource exhibited a significant protective effect. In terms of practical significance, within our sample, 7.2% (n = 66) of participants fell into the Total Vulnerability zone (*Z* < −1.255), representing students whose internal psychological resources—both career adaptability and JSSE—provide no significant buffering effect against employment anxiety. An additional 4.1% (*n* = 38) fell into the Single Protection zone (−1.255 < *Z* < −1.182), where only JSSE continues to offer protection. These figures suggest that approximately 11.3% of EFL college students in our sample may require targeted intervention beyond the enhancement of career adaptability alone. These findings suggest that a minimum level of proactive personality may be necessary for individuals to effectively draw on career adaptability and JSSE in mitigating employment anxiety.

## 5 Discussion

This study incorporates career adaptability, JSSE and proactive personality into the same analytical framework, and deeply explores the alleviation paths of employment anxiety among EFL college students from the perspective of SCCT. Based on the evidence-supported model, this section conducts a systematic analysis and discussion on the interaction mechanisms and internal logic among core variables.

For EFL college students, there is a significant negative correlation between career adaptability and employment anxiety. This finding is consistent with the research results of Fang and Xu (2025). According to SCCT (Lent et al., 1994), employment anxiety mainly stems from individuals’ uncertainty about the unknown occupational environment and their own competitiveness. Career adaptability is associated with a stronger sense of personal agency in professional growth and with greater capacity for managing challenges. These features also appear alongside lower anxiety levels related to labor market pressures. For EFL college students, this is especially relevant right now. With AI moving fast and machine translation getting better by the day, traditional language service jobs are changing, and changing fast. Under this situation, career adaptability becomes an important psychological resource for alleviating their AI anxiety (Wang, 2026). Students with strong career adaptability tend to be more proactive. They will actively collect employment information, adjust their phased goals according to market changes, and know how to mobilize the resources around them to cope with changes. This proactive attitude not only helps them quickly get out of negative emotions when facing job setbacks but also can, to a certain extent, enhance their subjective well-being (Ouyang et al., 2025). In the long run, the accumulation of career adaptability helps college students transform their career values into clear career decisions, enabling students to effectively cope with career uncertainties and pursue their self-actualization goals (Wang D. et al., 2025). Therefore, career adaptability can promote individual career development and lay a solid foundation for their mental health.

This study verified the mediating role of JSSE between career adaptability and employment anxiety. In other words, career adaptability can directly alleviate employment anxiety, and indirectly alleviate anxiety by enhancing the level of JSSE. This finding supports the core view of the SCCT (Lent et al., 1994) that an individual’s adaptive resources can enhance their belief in control of job-seeking tasks, thereby reducing the anxiety experience caused by uncertain situations. Specifically, students with strong career adaptability tend to be better at adjusting themselves according to the environment. This process of actively adapting helps them gain positive experiences more easily while job hunting and boosts their JSSE (Gerçek, 2024). Under the dual background of the continuous expansion of higher education enrollment and the in-depth adjustment of the economic structure in our country, the contradiction between supply and demand in the college student job market has become increasingly prominent, making graduates face more severe challenges in the job-hunting process. In this context, students who can continuously accumulate positive job search experiences and maintain high JSSE are more likely to break away from a mindset relying solely on language skills, actively expand their career positioning, and thus demonstrate stronger adaptability and competitiveness in the job market. In addition, when job hunting and facing pressure in the labor market, people with high JSSE tend to view difficulties as surmountable challenges rather than threats, and therefore experience less anxiety during their job search (Carkit, 2025). This study further deepens previous research, suggesting that educators can intervene in students’ anxiety by enhancing adaptability and job search confidence.

This study delves deeply into how proactive personality moderates the relationships of career adaptability, JSSE with employment anxiety, and the relevant hypotheses are supported. In simple terms, we found that in individuals with high proactivity, the buffering function of career adaptability and JSSE on employment anxiety would be more pronounced. One reason may be that people with high levels of proactivity tend to demonstrate a stronger motivation to achieve results. They tend to set career goals earlier and use different ways to prepare, thereby effectively converting their resources into practical actions to alleviate anxiety (Tu and Liu, 2025). Influenced by Confucian culture in China, concepts such as “perseverance in striving” and “preparing for rainy days” have actually been internalized in the value systems of many students. Those with high proactivity tend to receive more expectations from their families and society to “achieve early and become talented early.” This expectation will prompt them to prioritize career planning and accumulate competitive advantages through internships and participation in competitions. On the other hand, the situation for low proactivity students is different. They often do not tend to actively change the environment or actively respond to challenges. Even if they have certain adaptability or self-efficacy, they find it difficult to effectively transform them into employment preparation behaviors that alleviate anxiety (Ruan et al., 2025). These findings add to the current understanding of how proactive personality operates in this context. Although this study found that proactive personality significantly strengthened the buffering effect of career adaptability on employment anxiety and regulated the relationship between JSSE and employment anxiety as a protective factor, it did not play a moderating role between career adaptability and JSSE. This result is consistent with the findings of Meng et al. (2025), suggesting that proactive personality may play a more catalytic role rather than an amplifying role: it is better at activating external resources for the transformation to a positive psychological state, rather than directly intervening in the formation path of internal cognitive structures.

Our Johnson-Neyman analysis further revealed a tiered protection logic that carries critical implications for establishing clear intervention priorities. The results identified three distinct psychological defense states. In the “Double Protection” zone (*Z* > −1.182), both career adaptability and JSSE significantly buffer against employment anxiety. In the “Single Protection” zone (*Z* between −1.255 and −1.182), the buffering effect of career adaptability becomes non-significant, but JSSE continues to provide a protective function. In the “Total Vulnerability” zone (*Z* < −1.255), neither career adaptability nor JSSE exhibits a significant protective effect. Based on these findings, we argue that students in the “Total Vulnerability” zone must be the top priority for university counseling centers, as they cannot utilize any internal resources to manage employment-related stress. In our sample, 7.2% (*n* = 66) of participants fell into this zone. When bridging these findings with our demographic results, we note that this high-risk group is likely to overlap with the most anxious demographic groups, specifically senior students and females. This convergence suggests that female seniors with low proactive personality represent the most critically vulnerable subgroup requiring immediate and prioritized attention from university counseling services.

This study also found some pretty clear differences in employment anxiety among EFL college students when it came to gender and grade level. Look at gender, female students in this study reported more employment anxiety than male students. This lines up with what Li F. et al. (2025) and Yang et al. (2025) found in their research. One possible explanation is that when looking for work, women face more complex expectations regarding their gender roles and greater social pressure. Combined with their generally higher emotional sensitivity, they tend to perceive career uncertainty as a source of fear. In contrast, male students are more inclined to adopt positive coping strategies to deal with the emotional and stress challenges related to employment (Li C. et al., 2025). In a way, the gap in anxiety might actually be widened by these different coping styles. With regard to the grade, fourth-year students reported significantly higher anxiety than their peers in other years, a pattern also seen in Li L. et al. (2025). This outcome might stem from the particularity of the development stage it is facing: senior students stand at a critical juncture as they transition from campus to the workplace, and are confronted with multiple pressures such as graduation theses, internship evaluations, and job hunting. As application deadlines creep closer and the uncertainty about what comes next builds up, it’s only natural that their anxiety starts to climb. Unlike underclassmen, who are at the initial stages of career exploration, upperclassmen’s anxiety about job hunting stems largely from practical challenges related to competition in the job market and a direct desire for self-fulfillment.

## 6 Implications and limitations

### 6.1 Theoretical implication

This study started by looking at how career adaptability relates to employment anxiety among EFL college students. The main takeaway is that students who are more adaptable tend to feel less anxious about employment. The findings also revealed an underlying mediating mechanism: career adaptability not only directly alleviated employment anxiety among EFL college students but also operated indirectly by enhancing their JSSE. In addition, proactive personality traits amplified the benefits of both career adaptability and JSSE on reducing employment anxiety. This study found that the groups differed in terms of employment anxiety, with these differences depending on gender and grade. This provided empirical data for a deeper understanding of the psychological adjustment patterns of this population group. At the theoretical level, these findings advance our understanding of how academic background interacts with employment pressure, contributing to the development of more targeted analytical frameworks for examining employment-related psychology. Based on the unique professional learning ecosystem and employment context of EFL college students, this study applies the classic social cognitive career theory to a more specific group, testing and expanding the scope of application of this theory. The conclusions deepen our understanding of the interactive mechanisms among career adaptability, self-efficacy beliefs, and personality traits, offering both a novel explanatory framework and empirical support for career development research in specific disciplinary contexts. These findings add something to how we think about employment anxiety. It also lays the groundwork for future research on the interaction between occupational characteristics and work-related psychological mechanisms.

### 6.2 Practical implication

Here are some practical steps that may help EFL college students reduce employment anxiety and better prepare EFL college students for the job market. EFL college students should actively overcome the traditional role constraints of their first years of study and develop a forward-looking attitude toward their professional development. By actively participating in lectures on the latest industry developments, completing in-depth internships at companies, and engaging in other practical activities, they can gain a better understanding of new professional fields and career paths, as well as reduce the uncertainty associated with job hunting that arises from the difficulty of gathering information and processing the knowledge we have acquired. Additionally, students are encouraged to integrate job preparation into their daily learning processes by proactively undertaking practical exercises such as resume writing and mock interviews. Thanks to these numerous small successes, they can gradually develop a sense of professional independence, which facilitates a subjective shift from passive waiting to actively shaping their own destiny.

EFL educators should expand their traditional instructional roles, evolving from mere language transmitters into career development facilitators. In course design, the embedding of real workplace cases and the design of project-based learning tasks should be strengthened, enabling students to acquire professional knowledge while forming a systematic understanding of the workplace ecosystem. Furthermore, for students who suffer from excessive anxiety or a tendency toward self-criticism, timely intervention and collaboration with university counseling centers are essential. Such measures can prevent employment anxiety from evolving into deep-seated psychological distress, thereby achieving an organic integration of knowledge imparting and emotional support.

Colleges and universities should respond to the structural tension between talent cultivation and market demand at the institutional level and promote the systematic optimization of the curriculum system and education model. During the lower grade years, educational institutions should step up their efforts in career guidance by systematically familiarizing students with various industries and organizing hands-on workplace experiences to break down information barriers. For upper-year students, the focus should shift to enhancing job-seeking skills and simulating authentic workplace scenarios to improve employment adaptability. Even more importantly, universities should create an educational environment that fosters curiosity and encourages experimentation. Through various channels such as outstanding alumni sharing, phased task design, participation in scientific research projects, and innovation and entrepreneurship competitions, students’ abilities to actively obtain information and independently plan their development are cultivated.

### 6.3 Limitations

Based on the research results and discussions, this study still has some limitations, which need to be further explored and improved in future research. First, the data is collected at a specific point in time. This means that we cannot draw definitive conclusions about cause-and-effect relationships, but only about how these variables are related to one another. Future research can conduct multi-time point data collection at different key nodes of college students’ job hunting to more accurately reveal the dynamic evolution process and causal direction among variables. Second, the sample only included EFL college students, and was recruited through convenience sampling. The language professional background and specific career path choices of this group have certain particularities, and the convenience sampling approach may limit the generalizability of our findings to broader student populations. Despite our statistical adjustment for gender, age, and grade across all analyses to reduce possible selection bias, subsequent investigations would benefit from broader sampling that covers students from varied academic disciplines, institution types, and geographic regions. More rigorous sampling procedures, such as probability-based approaches, could also be employed to examine whether the current patterns hold across different population subgroups. Third, this study failed to incorporate remote situational factors such as social support, family background and macroeconomic environment that affect an individual’s career development into its investigation. Future studies could build more complex models that bring in these factors, to better capture the full picture of what’s going on beneath the surface. Fourth, while prior research suggests that students’ current career or work status, including internship experience or secured job offers, may influence their levels of job search self-efficacy and employment anxiety, this study did not include these variables in the survey design. Consequently, we were unable to control for or examine the potential effects of prior professional exposure. Future research should incorporate comprehensive measures of internship experience, including quantity, quality, relevance to majors, and duration, as well as job offer status, to more fully capture the role of career exposure in shaping employment related psychological outcomes. Fifth, our PROCESS analysis treats composite scores as observed variables without modeling item-level measurement error. Although CFA confirmed strong psychometric properties of all scales, future research may employ full SEM to cross-validate our findings and account for measurement error more comprehensively.

## 7 Conclusion

Based on SCCT, this study systematically analyzed the internal mechanism between career adaptability and employment anxiety among EFL college students. In addition, the mediating effect of JSSE and the moderating effect of a proactive personality were examined. The findings revealed that career adaptability correlated negatively and significantly with employment anxiety among EFL college students. JSSE partially mediated this relationship. Proactive personality moderated both the relationship between career adaptability and employment anxiety and the relationship between JSSE and employment anxiety. All theoretical hypotheses proposed in this study received empirical support. These results carry practical relevance for career counseling and psychological support services within college and university settings. However, since all variable data in this study were measured at one time point, the relationships among the variables remain correlational in nature, and therefore, causal relationships cannot be inferred. Future research can adopt long-term tracking designs or conduct interventional experiments to more clearly reveal the sequence and long-term interaction mechanisms among various variables.

## Data Availability

The raw data supporting the conclusions of this article will be made available by the authors, without undue reservation.

## Footnotes

1
https://www.sojump.com/
